# Deep fake detection using a sparse auto encoder with a graph capsule dual graph CNN

**DOI:** 10.7717/peerj-cs.953

**Published:** 2022-05-31

**Authors:** Venkatachalam Kandasamy, Štěpán Hubálovský, Pavel Trojovský

**Affiliations:** 1 Department of Applied Cybernetics, Faculty of Science, University of Hradec Králové, Czech Republic; 2 Department of Mathematics, University of Hradec Králové, Hradec Králové, Czech Republic

**Keywords:** DeepFake, Deep learning, Generative adversarial networks, Long short term memory (LSTM), Graph LSTM, Capsule convolution neural network

## Abstract

Deepfake (DF) is a kind of forged image or video that is developed to spread misinformation and facilitate vulnerabilities to privacy hacking and truth masking with advanced technologies, including deep learning and artificial intelligence with trained algorithms. This kind of multimedia manipulation, such as changing facial expressions or speech, can be used for a variety of purposes to spread misinformation or exploitation. This kind of multimedia manipulation, such as changing facial expressions or speech, can be used for a variety of purposes to spread misinformation or exploitation. With the recent advancement of generative adversarial networks (GANs) in deep learning models, DF has become an essential part of social media. To detect forged video and images, numerous methods have been developed, and those methods are focused on a particular domain and obsolete in the case of new attacks/threats. Hence, a novel method needs to be developed to tackle new attacks. The method introduced in this article can detect various types of spoofs of images and videos that are computationally generated using deep learning models, such as variants of long short-term memory and convolutional neural networks. The first phase of this proposed work extracts the feature frames from the forged video/image using a sparse autoencoder with a graph long short-term memory (SAE-GLSTM) method at training time. The first phase of this proposed work extracts the feature frames from the forged video/image using a sparse autoencoder with a graph long short-term memory (SAE-GLSTM) method at training time. The proposed DF detection model is tested using the FFHQ database, 100K-Faces, Celeb-DF (V2) and WildDeepfake. The evaluated results show the effectiveness of the proposed method.

## Introduction

With the advancement of technology, accessibility to social networks is easier for all users. Therefore, many deepfake images and videos have been spread on social media platforms. Manipulation of digital images or videos on social media involves replacing the image of a person with the face of another person. This manipulation of facial images is called deepfake and has become a very annoying social problem nowadays. Swapping popular faces with celebrities from Hollywood or politicians will mislead people’s opinions and create rumors about celebrities or politicians (Wang et al., 2020; Nataraj et al., 2019). The spread of false information in the form of deepfake through synthetically created images and videos has increased daily. This will become a significant issue for manipulative detection techniques.

Detection and prevention of deepfake images and videos are essential on social media. For these research studies, various organizations, such as Facebook Inc., the US Defense Advanced Research Projects Agency (DARPA), and Google, support researchers in detecting and preventing deepfake images and videos. (Westerlund, 2019; Kwok & Koh, 2021). In the detection of forged images and videos, many research works have been developed, and these research works focus on keeping personality information secret in a secure way. To detect the difference between real and fake images, ocular biometrics, based on the CNN approaches of SqueezeNet, DenseNet, ResNet and light CNN (Nguyen, Yamagishi & Echizen, 2019), is used. To ensure personnel data security and avoid the deepfakes, the researchers are motivated to develop an efficient deepfake detection system using deep learning approaches. The main contributions of this research work are as follows.

1. Implementing the deepfake face detection method based on the SAE-GLSTM and capsule dual graph CNN model in which dimensionality reduction is used for the features in the face image.
2. To improve accuracy, preprocessing in this work implements an adaptive median filter and uses GLSTM to extract image features.
3. Compare to the existing research works, our proposed is efficient in deepfake detection system using deep learning based preprocessing, feature extraction and detection. This system ensures the efficiency, reliability and integrity.

The article is organized as follows. “Review of Literature” describes a review of the literature, “Proposed Methodology” introduces deep detection using SAE-GLSTM and the capsule dual graph CNN model, “Experimental Result & Discussions” discusses the experimental results, and “Conclusion” concludes the article with future directions.

## Review of Literature

Deepfake is composed of “deep learning” and “fake” concepts with a technique for synthesizing videos or images using deep learning techniques. Deepfake enthusiasts swap the face of an image or perform video forgery to spread misinformation, mask real information, and promote privacy insecurity using advanced techniques such as artificial intelligence and deep learning techniques. This swapping of images or videos of faces has become an annoyance for social media platforms. Social media users have had difficulty publishing forged images or videos that are developed by combining celebrity or politician images in the video (Kaur, Kumar & Kumaraguru, 2020).

Detection of face tampering using the CNN model is based on the concept of two streams of face classification and patch triplets. In the training process, the face image was classified as having been tampered with (Zhou et al., 2017). One article (Korshunova et al., 2017) proposed the transformation of the original face image into a fake image using a face-swap process. However, it preserves facial expressions, lighting, and position of images from the source to the destination of the image. The first deepfake video was released in 2017 in which a celebrity face was swapped with the face of a porn actor. Additionally, deepfake videos of famous politicians were created as fake speeches. It is a great threat to the world for the preservation of the security and privacy of world-famous actors and politicians (Howcroft, 2018; Chesney & Citron, 2019; Soare & Burton, 2020). Identifying the original face image from the deepfake video is evaluated based on the properties of deepfake videos, which undergo an affine warping process to match the face of the original image (Li & Lyu, 2019).

Fake face videos are exposed using deep-generative networks based on eye blinking features. To achieve this, it detects the faces frame by frame and aligns them with the same coordinate value and observes head movement in a realistic manner (Li, Chang & Lyu, 2018). From each frame of a video, eye blinking is detected using the long-term recurrent convolutional neural network (LRCN) method. In this LRCN method, the swapping of the face in the videos is implemented more easily. A pipeline-based temporal-aware system was proposed to detect deepfake videos automatically. Features are extracted in the form of a frame by a frame (Güera & Delp, 2018). Table 1 shows the results of a survey of the deepfake detection methods.

**Table 1:** Survey on deepfake detection methods.

Author	Classifier	Type of input	Dataset
Hsu, Zhuang & Lee (2020)	CNN concatenated to CFFN	Image	CelebA, DCGAN WGAN WGAN-GP, least squares GAN PGGAN.
Chintha et al. (2020)	Convolutional bidirectional recurrent LSTM network	Videos	FaceForensics++ and Celeb-DF (5,639 deepfake videos) and the ASVSpoof Access audio dataset.
Agarwal et al. (2020)	CNN	Videos	Four in-the-wild lip-sync deep fakes from Instagram and YouTube (www.instagram.com/bill posters ukand youtu.be/VWMEDacz3L4).
Fernandes et al. (2020)	ResNet50model [102], pretrained on VGGFace2	Videos	VidTIMIT and two other original datasets obtained from the COHFACE and Deepfake TIMIT datasets.
Sabir et al. (2019)	Spatiotemporal features with RCN	Videos	FaceForensics++ dataset, including 1,000 videos.
Xuan et al. (2019)	DCGAN, WGAN-GP and PGGAN.	Images	CelebA-HQ, DCGAN, GAN-GP and PGGAN
Yang, Li & Lyu (2019)	SVM	Videos/Images	UADFV consists of 49 deepfake videos, and 252 deepfake images from DARPA MediFor GAN Image/Video Challenge.
Nguyen, Yamagishi & Echizen (2019)	Capsule networks	Videos/Images	The Idiap Research Institute replayattack, facial reenactment FaceForensics.
Afchar et al. (2018)	CNN	Videos	Deepfake one constituted from onlinevideos and the FaceForensics one created by the Face2Face approach.
Güera & Delp (2018)	CNN and LSTM	Videos	A collection of 600 videos obtained from multiple websites.
Li, Chang & Lyu (2018), Li & Lyu (2019)	LRCN	Videos	Consists of 49 interview and presentation videos, and their corresponding generated deepfakes.

## Proposed Methodology

**Figure 1:**
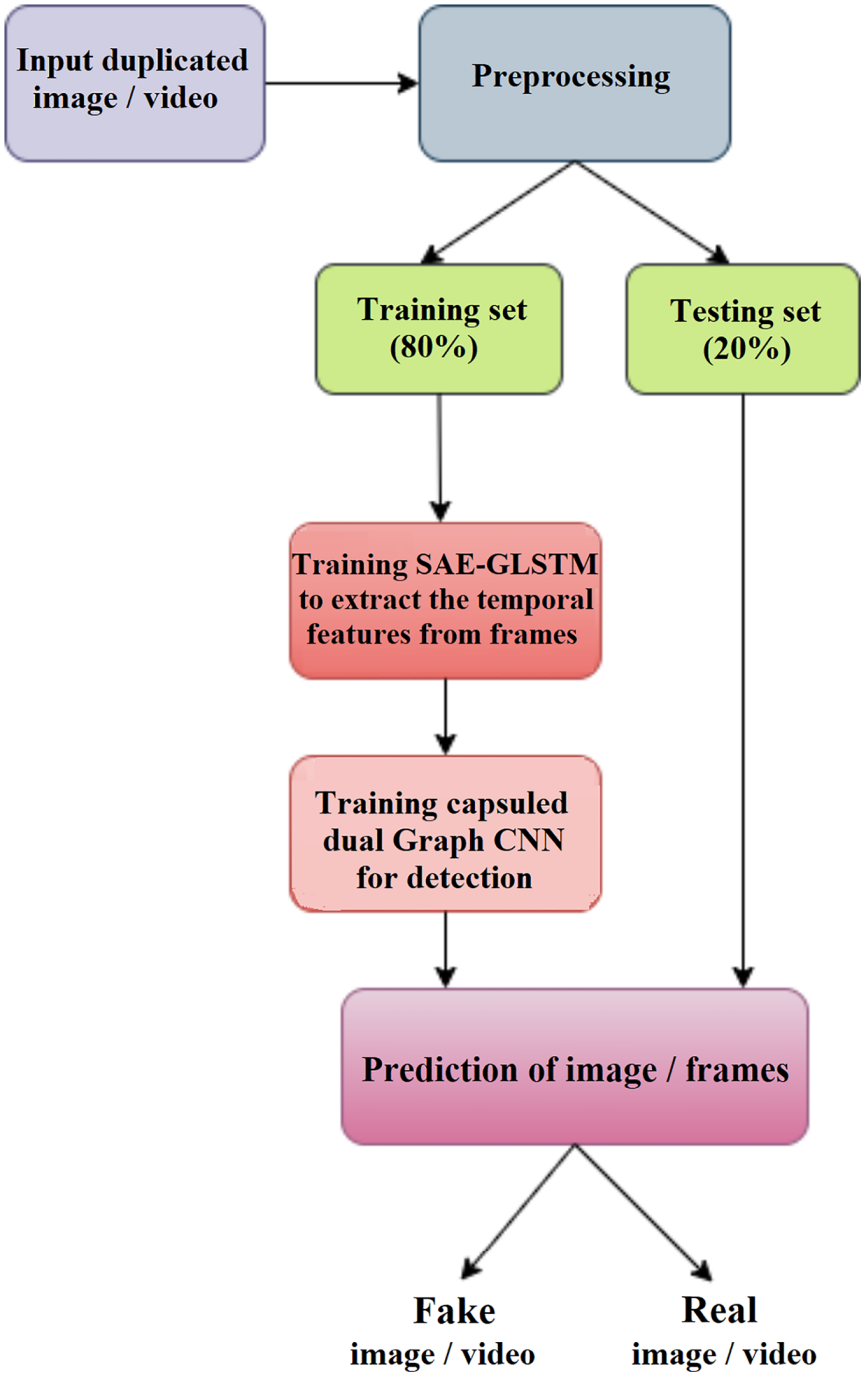
Overview of proposed DeepFake detection system.

The proposed deepfake detection method shown in Fig. 1 is suitable for video and images. In the pre-processing phase, the deepfake video input is split into frames, and detection is performed from the frames. During the training phase, the features of the video frames or images are extracted using the graph LSTM method. The extracted feature subset is given as input to phase II for detection using a capsule network, the capsule dual graph CNN, which identifies the frame sequence/image as real or fake.

### Preprocessing

**Figure 2:**
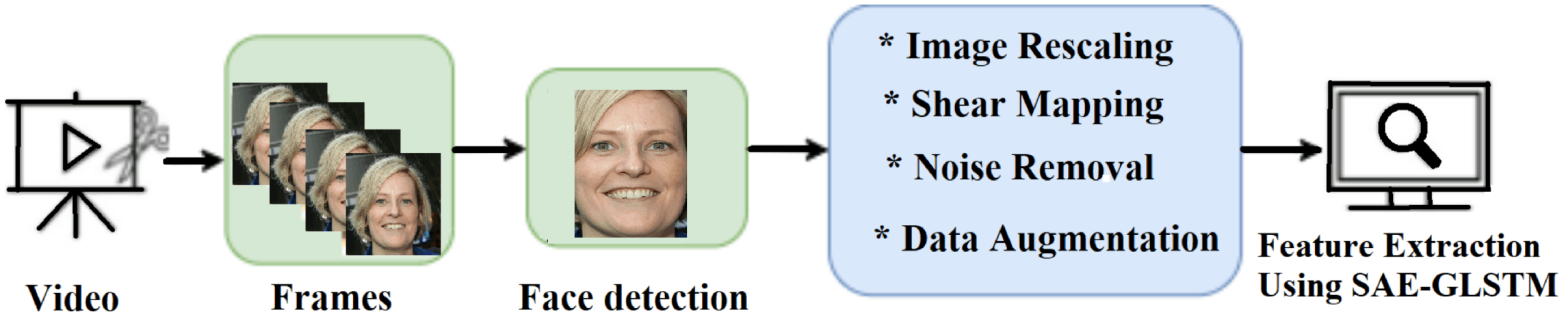
Proposed stages of preprocessing.

The preprocessing of fake videos/images is shown in Fig. 2. Initially, the input fake videos are split into frames. Using multitask cascaded convolutional neural networks (MTCNNs) (Zhang et al., 2016), the face is detected and cropped from video frames. MTCNN is a Python face detection module with an accuracy of 95%. It can extract the face that focuses on computer vision transformation. This extracted face is pre-processed using the sequence of operations listed below to enhance image quality. The feature vector was generated by extracting the computer vision features from the preprocessed image using the graph LSTM method.

#### Image rescaling

The input image/frames consist of RGB values in the range of 0 to 255. The values are rescaled to the interval [0,1] to be fed as input into the proposed model using the 1/255 scaling method.

#### Shear mapping

Each image in the frames is converted from the edge to the vertical direction. From the original frame, this parameter controls the angle of deviation of the horizontal line and the displacement rate. The value of the shear range is 0.3.

#### Noise removal

Accurate noise removal of input data will build up the improved quality training data set. This will improve the accuracy of the detection system. The background noise of the normalized image is removed using an adaptive median filter. An adaptive median filter solves issues related to median filters, such as the capability of the median filter to remove only salt and pepper noise. If there is no proper kernel size smoothing and if the spatial density is high, then the median filter is not effective. Various adaptive median filters are suitable for kernels with variable sizes, and all pixels are not replaced with median values. In Algorithm 1, we follow Soni & Sankhe (2019), in which the idea of a two-level median filter appeared. This algoritm consists of two steps. For each kernel, the median value is calculated and the pixel value of salt and pepper noise is checked. We consider the input grayscale image with pixel dimensions *m* × *n*. We assume that this image is given as the matrix *G* of the gray levels of all pixels in the image, so *G* = (*g*_*x*,*y*_), where $x = 1, \ldots ,m$, $y = 1, \ldots ,n$, and *g*_*x*,*y*_ is a gray level of the pixel in the coordinates (*x*,*y*). Algorithm 1 will transform the input gray level *g*_*x*,*y*_ of the pixel in the coordinates (*x*,*y*) into the noise removed value of the gray level of this pixel. The method of transformation of the value *g*_*x*,*y*_ is done in the construction of all *s*-neighborhoods *U*_*s*_ of the pixel in coordinates (*x*,*y*) (this pixel lies in the center of these squre-neighborhoods) with the side of this square equal to *s* (the initial value is *s* = 3) and we manage this transformation in two levels *L*_*A*_ and *L*_*B*_, in which we compare the value *g*_*x*,*y*_ with the minimal, maximal, and median grayscale values of all pixels in neighborhood *U*_*s*_ for *s* ≥ 3. The exact procedure of Algorithm 1 is evident from the pseudocode below; only we must realize that for the smallest *s*-neighborhood with the side *s* = 3, the pixel inside the image has eight adjacent pixels, but the pixels in the corners have only three neighbors, and the other pixels at the edge of the image have five neighbors.

**Algorithm 1:** Two-Level Adaptive Median Noise Removal Filter.

**Input:** The gray level *g*_*x*,*y*_ of the pixel at the coordinates (*x*,*y*), the maximal value *s*_*max*_ of the side of the square *U*_*s*_.
**Output:** The noise removed value of the gray level ${\overline g _{x,y}}$ of the pixel in the coordinates (*x*,*y*).
Step 1: Assume that *L*_*A*_ and *L*_*B*_ are the two levels of noise pollution.
Step 2: Consider a *s*-neighborhood *U*_*s*_. Initially, we take *s* = 3.
Step 3: Calculate *g*_*med*_, *g*_*min*_, *g*_*max*_ as the median, minimal, and maximal gray level of the pixels in *U*_*s*_.
Step 4: Level *L*_*A*_.
${A_{11}} = {g_{med}} - {g_{min}},\quad {A_{12}} = {g_{med}} - {g_{max}}.$
Step 5: **if** (*A*_11_ > 0 && *A*_12_ < 0) **then** go to Step 11.
Step 6: **else** *s* = *s* + 2 // We increase the size of *s*-neighborhood *U*_*s*_ (*s* must be an odd number, as the pixel (*x*,*y*) must be in the center).
Step 7: **end if**
Step 8: **if** *s* ≤ *s*_*max*_ **then** go to Step 4.
Step 9: **else** Output: ${\overline g _{x,y}} = {g_{x,y}}$.
Step 10: **end if**
Step 11: Level *L*_*B*_.
${B_{11}} = {g_{x,y}} - {g_{min}},\quad {B_{12}} = {g_{x,y}} - {g_{max}}.$
Step 12: **if** (*B*_11_ ≥ 0 && *B*_12_ ≤ 0) **then** Output: ${\overline g _{x,y}} = {g_{med}}$.
Step 13: **else** Output: ${\overline g _{x,y}} = {g_{x,y}}$.
Step 14: **end if**

#### Data augmentation

During the training process, the augmentation method is as follows:

1. *Zooming augmentation:* zooming augmentation is used to view the input image as larger with the value 0.2 in the range [0.8, 0.2]. The parameter values vary from the 1 − value to the 1 + value.
2. *Horizontal flipping:* with the help of Boolean value ‘true’, the zoomed image is horizontally flipped.
3. Random rotation of approximately 30°.
4. Random contrast, brightness and saturation jitter.
5. Coarse dropout with the size of 0.03.

### Feature extraction using the sparse autoencoder (SAE) with graph LSTM

The pre-processed image is fed into this section to extract the computer vision features using the proposed sparse autoencoder-based LSTM model. The traditional auto-encoder method has some problems, such as its inability to find features by copying memory into an implicit layer (Olshausen & Field, 1996). This problem is resolved with a sparsity approach with an autoencoder called a sparse autoencoder (SAE) (Hoq, Uddin & Park, 2021). It is an unsupervised deep learning method with a single hidden layer (Kang et al., 2017) used to encode the data for feature extraction. This will extract the most relevant features from the expressions of the hidden layer and estimate the error (Leng & Jiang, 2016). The regularization equation for the sparsity is defined in Eq. (1).

(1) $${Sparsity\_regrsn} = \sum\limits_{i = 1}^{{u_2}} KL(\vartheta ||{\widehat \vartheta _i}),$$

where the divergence implemented is the Kullback-Leibler divergence (KL), ${\widehat \vartheta _i}$ is the activation function of the hidden node *i*^*th*^, *ϑ* is the sparsity parameter. The KL-divergence is mathematically defined by Eq. (2)

(2) $$KL(\vartheta ||{\widehat \vartheta _i}) = \vartheta \log \displaystyle{\vartheta \over {{{\widehat \vartheta }_i}}} + \left( {1 - \vartheta } \right)\log \displaystyle{{1 - \vartheta } \over {1 - {{\widehat \vartheta }_i}}}.$$

**Figure 3:**
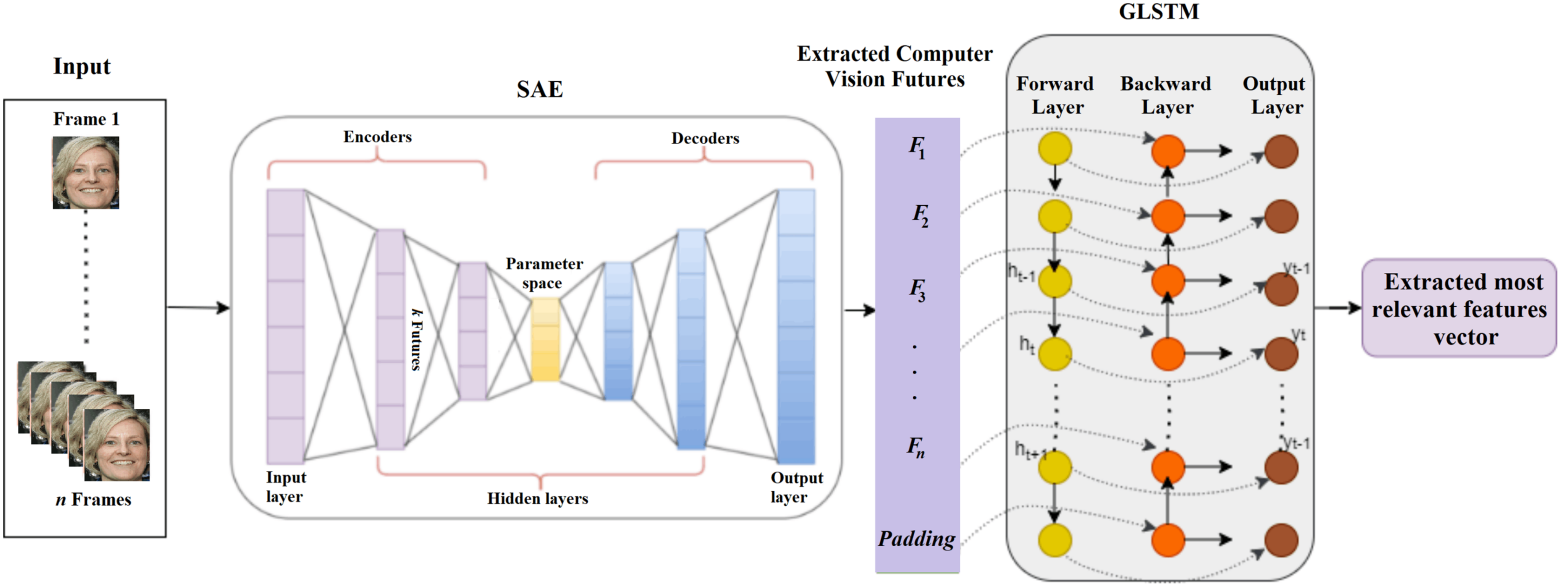
Deep fake image feature extraction using proposed SAE-GLSTM network.

The sparse AE is trained with the cost function declared in Eq. (3) which consists of the mean square error defined in Eq. (4). This will reconstruct the input vector *X* into the output vector $\widehat X$ throughout the entire training dataset (Ng, 2011). The LASSO regression term is declared in Eq. (3) and the final term of the sparsity transformation is defined in Eq. (1). The importance of LASSO regression in SAE is to extract the most relevant features by assigning the feature coefficients as zero for features that are of little relevance, which will reduce the space of the parameter.

(3) $${Cost\_function} = {MSE + \alpha \cdot Lasso\_\;regrsn + \beta \cdot Sparsity\_regrsn,}$$

(4) $$MSE = \displaystyle{1 \over N}\sum\limits_{i = 1}^N {({X_i} - {\hat X_i})^2},$$

(5) $$Lasso\_regrsn = \sum\limits_{l = 1}^{{n_l} - 1} \sum\limits_{i = 1}^{{u_l}} \sum\limits_{j = 1}^{{u_l} + 1} |w_{ij}^l|,$$

where *N* is the total number of input data points, *X* represents the input vector, $\hat X$ represents the reconstructed output vector, *α* is the Lasso regression coefficient and *β* is the sparsity regularization coefficient, *l* indicates the *l*^*th*^ layer, *n*_*l*_ is the number of layers, *u*_*l*_ is the number of units in the layer *l* and *w*^*l*^_*ij*_ is the weight value between the *i*^*th*^ node of the layer *l* and the *j*^*th*^ node of the layer *l* + 1. LASSO regression can add the magnitude of the absolute value as a penalty term. To improve the feature extraction process, this penalty coefficient is set to zero. The LSTM trains SAE to improve the feature extraction process. An LSTM is a backpropagation method for training the feature extraction model with three gates: input, forget, and output gates. The input gate is responsible for modifying the memory with the values decided by the sigmoid activation function. The forget gate is used to discard features from the previous state. The output gate is used to control the output features. Traditional LSTM is enhanced with a graph structure in which each node of the graph is represented as a single LSTM unit with forward and backward directions. In one direction, the node history is constructed and in another direction the response is characterized. Figure 3 shows the proposed SAE-GLSTM model to extract deepfake image features.

**Figure 4:**
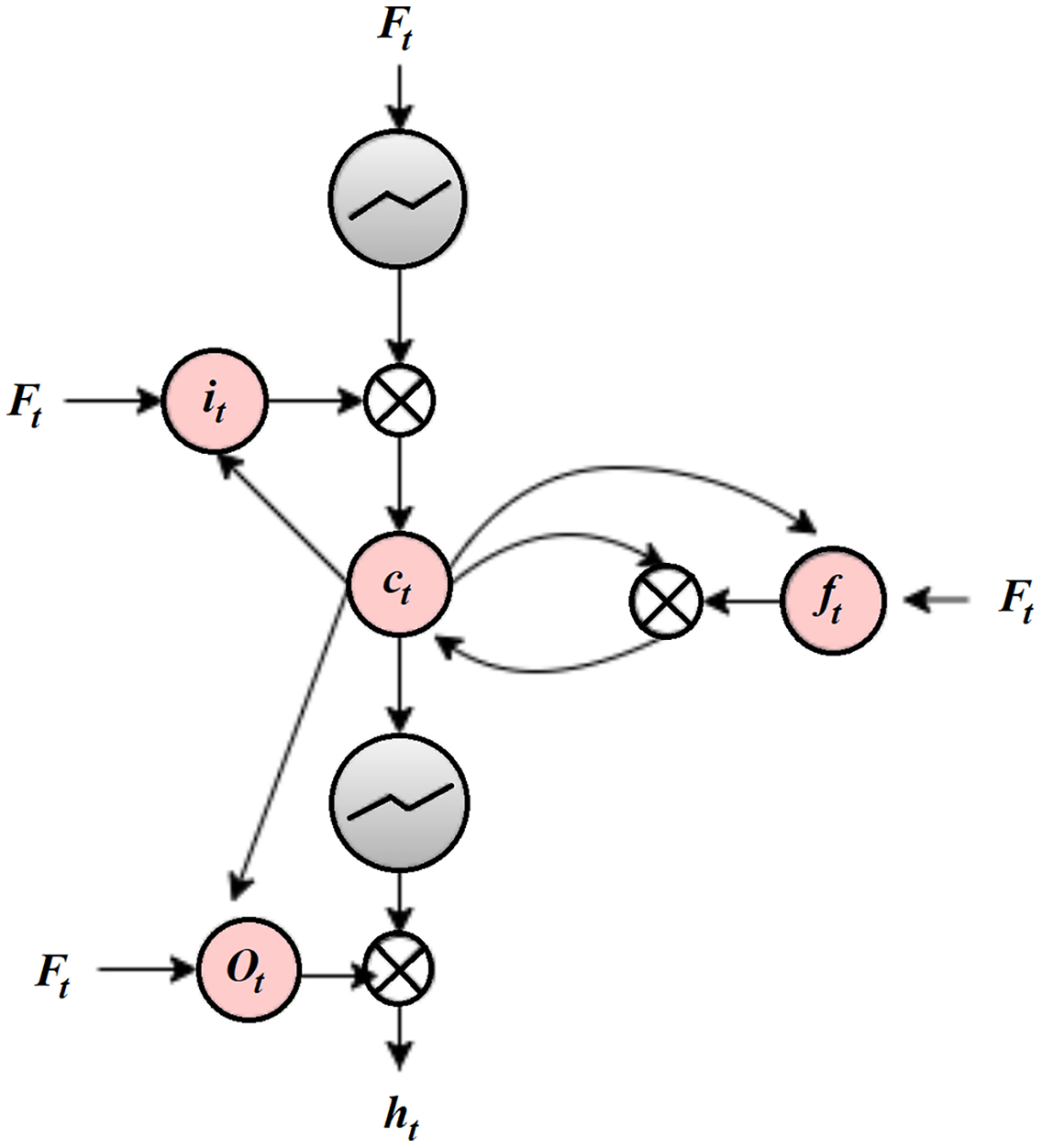
GLSTM cell.

An SAE consists of an encoder, a compression/parameter space, and a decoder. The encoder encodes the given input to the parameter space through hidden layers, and the decoder is responsible for decoding the parameter space data to the output layer. Due to the autoencoder nature of dealing with negative values, a rectified linear unit (ReLU) is not suitable, and a sigmoid function is used as an activation function, which will reduce the training ability of the network. SAE can reduce the error between the input and the reconstructed data. The usage of hidden layers is reduced by the sparsity constraint. The regularization used here will avoid the overfitting issue and can be applied to larger datasets. The selected features are then passed on to the LSTM cell to enhance the feature selection process by extracting the relevant features. The LSTM graph model comprises six layers: the input layer, four hidden layers, and the output layer. The input layer of the LSTM graph consists of the features extracted from SAE. Each feature is represented as a neuron in the LSTM graph cell input layer graph, as shown in Fig. 4.

**Figure 5:**
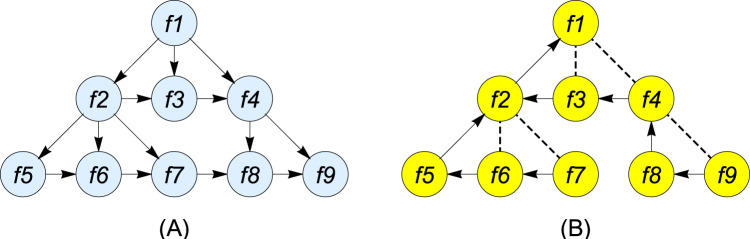
Hierarchical timing structure of (A) forward GLSTM (B) backward GLSTM.

For the number of iterations *t*, SAE-GLSTM computes the hidden forward sequence as $\overleftarrow s$ and the hidden backward sequence as $\overrightarrow s$, and the output sequence is represented as *Y*. The input *x*_*t*_ has a hierarchical timing layer and the transition of the node state is declared a vector using the standard LSTM (Zhou, Xiang & Huang, 2020). Figure 5 shows the hierarchical forward and backward timing structure of Graph LSTM.

Let *p*(*t*) be considered the parent node of *t* and *k*(*t*) be the first child node. This hierarchical timing structure has predecessor *p*(*t*) and successor *s*(*t*) representing the forward and backward siblings, respectively. If there is no child, the values of *k*(*t*), *p*(*t*), and *s*(*t*) are set to null. For the forward GLSTM, the parameters, such as the input gate *ig*, temporal forget gate *fg*, hierarchical forget gate *hg*, cell *c*, and the output *op*, are updated and represented in Eqs. (6) to (10) with the vectors *ig*_*t*_ indicating the new information weight, *fg*_*t*_ indicating the memory data of siblings, *hg*_*t*_ indicating the memory data of the parents, and *σ* representing the sigmoid function. For the backward GLSTM, $\mu \left( t \right)$ is replaced by *k*(*t*), *p*(*t*) is replaced by *s*(*t*) and is represented in Eqs. (11) to (15) listed in Table 2.

**Table 2:** Forward and backward sequence equations of the graph LSTM.

Forward sequence	Backward sequence
(6) $i{g_t} = \sigma ({w_{ig}}{x_t} + {U_{ig}}{h_{p\left( t \right)}} + {V_{ig}}{h_{\mu \left( t \right)}} + {b_{ig}})$	(11) $i{g_t} = \sigma ({w_{ig}}{x_t} + {U_{ig}}{h_{s\left( t \right)}} + {V_{ig}}{h_{k\left( t \right)}} + {b_{ig}})$
(7) $f{g_t} = \sigma ({w_{fg}}{x_t} + {U_{fg}}{h_{p\left( t \right)}} + {V_{fg}}{h_{\mu \left( t \right)}} + {b_{fg}})$	(12) $f{g_t} = \sigma ({w_{fg}}{x_t} + {U_{fg}}{h_{s\left( t \right)}} + {V_{fg}}{h_{k\left( t \right)}} + {b_{fg}})$
(8) $h{g_t} = \sigma ({w_{hg}}{x_t} + {U_{hg}}{h_{p\left( t \right)}} + {V_{hg}}{h_{\mu \left( t \right)}} + {b_{hg}})$	(13) $h{g_t} = \sigma ({w_{hg}}{x_t} + {U_{hg}}{h_{s\left( t \right)}} + {V_{hg}}{h_{k\left( t \right)}} + {b_{hg}})$
(9) ${c_t} = {w_c}{x_t} + {U_c}{h_{p\left( t \right)}} + {V_c}{h_{\mu \left( t \right)}} + {b_c}$	(14) ${c_t} = {w_c}{x_t} + {U_c}{h_{s\left( t \right)}} + {V_c}{h_{k\left( t \right)}} + {b_c}$
(10) $o{p_t} = \sigma ({w_{op}}{x_t} + {U_{op}}{h_{p\left( t \right)}} + {V_{op}}{h_{\mu \left( t \right)}} + {b_{op}})$	(15) $o{p_t} = \sigma ({w_{op}}{x_t} + {U_{op}}{h_{s\left( t \right)}} + {V_{op}}{h_{k\left( t \right)}} + {b_{op}})$

The extracted features from SAE are enhanced with the LSTM graph by sending the SAE output as input to the LSTM unit. The optimal relevant feature subset has been selected as an output of this graph LSTM network. The extracted feature subset has numerical values of specific images in matrix form.

### Deepfake detection using capsule dual graph CNN

The proposed detection system consists of three capsules. Two capsules are allotted for output to indicate fake and real images. CNN dual graph is represented in one capsule to perform detection. The features extracted from “Feature Extraction using the Sparse Autoencoder (SAE) with Graph LSTM” are given as input to this detection model. The dual graph neural network is the variance of the traditional neural network with a graph (Scarselli et al., 2008). Each node in the graph is a feature. A dual graph CNN consists of two CNNs and the input set of data points $\mathcal{X} = \left\{ {{x_1},{x_2}, \ldots ,{x_l},{x_{l + 1}}, \ldots ,{x_n}} \right\}$, the set of labels $\mathcal{C} = \{ 1,2, \ldots ,c\}$, the first *l* points have labels $\{ {y_1},{y_2}, \ldots ,{y_l}\} \in \mathcal{C}$, and a graph structure. We assume that each point has at most *k* features; therefore, we denote the data set as a matrix $X \in {{\rm {\mathbb R}}^{n \times k}}$ and represent the structure of the graph by the adjacency matrix $A \in {{\rm {\mathbb R}}^{n \times n}}$. Using the input *X*, labels *L*, and *A*, our model aims to predict the labels of the unlabeled points.

**Figure 6:**
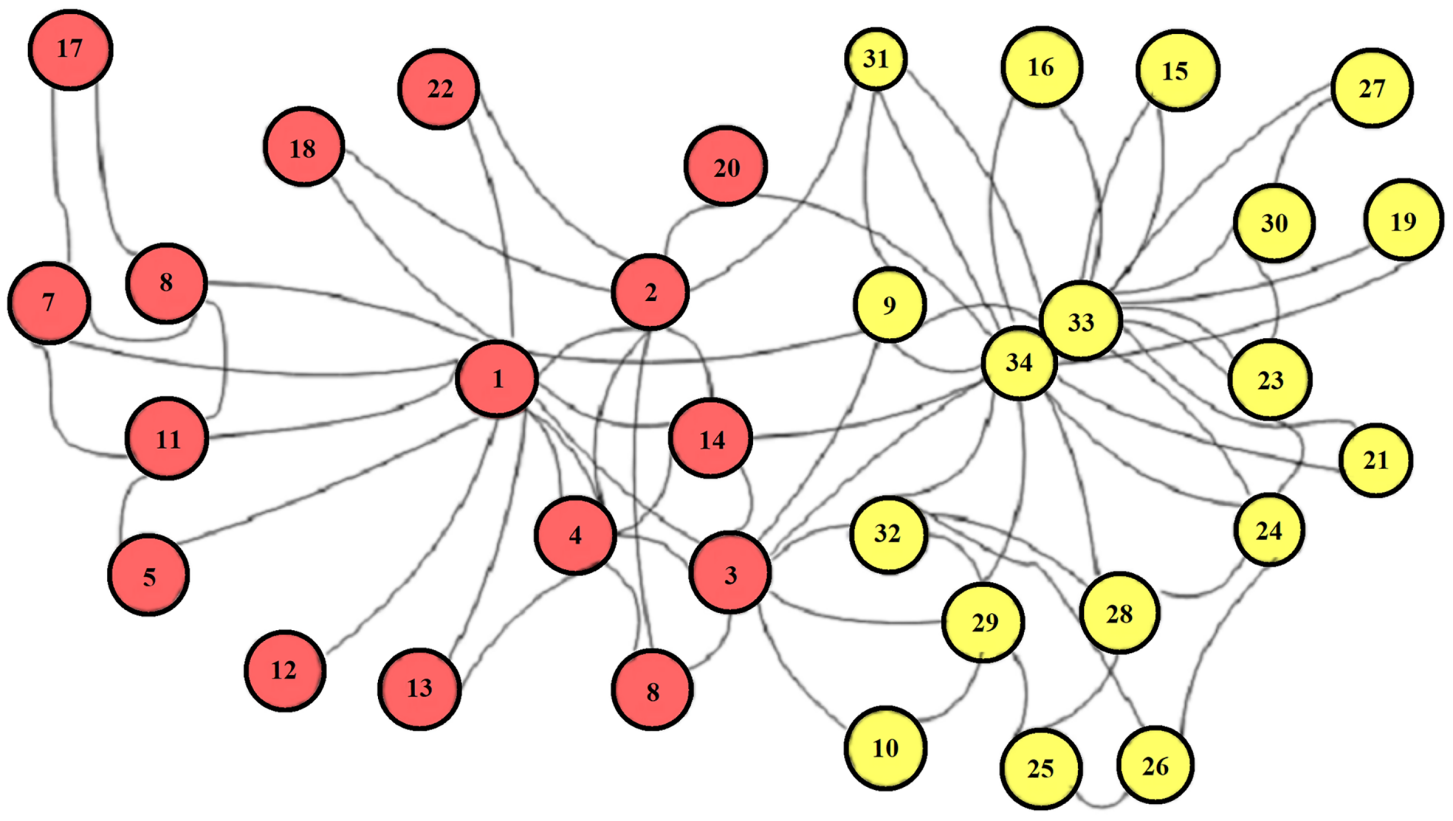
Karate club network for LC convolution (Kipf & Welling, 2017).

The model is constructed with local consistency (LC) and is a type of feed-forward network that incorporates global consistency (GC) and a regularizer for the ensemble. The feature vector FV and the adjacency matrix *A* are the inputs of the DGCNN model. The local consistency output for the hidden layer *i* of the network *Z*^(*i*)^ is declared in Eq. (16) (Kipf & Welling, 2017)

(16) $$conv_{LC}^{(i)}\left( X \right) = {Z^{(i)}} = \sigma \left( {{{\overline D }^{ - \textstyle{1 \over 2}}}\overline A {\kern 1pt} {{\overline D }^{ - \textstyle{1 \over 2}}}{Z^{(i - 1)}}{W^{(i)}}} \right),$$

where $\overline A = A + {I_n}$ is the adjacency matrix *A* with self-loops, *I*_*n*_ is the identity matrix, and ${\overline D _{i,i}} = \sum\nolimits_j {\overline A _{i,j}}$. Therefore, ${\overline D ^{ - \textstyle{1 \over 2}}}\overline A {\overline D ^{ - \textstyle{1 \over 2}}}$ is the normalized adjacency matrix, *Z*^(*i*−1)^ represents the output of the (*i* − 1)^*th*^ layer, *Z*^(0)^ = *X W*^(*i*)^ represents trainable parameters of the network, and *σ* indicates the activation function (ReLU). The output of the DGCNN can be visualized on the Karate club network, as shown in Fig. 6. The red color of this network indicates a labeled node and the green color indicates an unlabeled node. The local consistency network is optimized with PPMI (positive point-wise information) in the global consistency layer.

Global consistency is formed with PPMI to encode semantic information and is denoted as a matrix $P \in {{\rm {\mathbb R}}^{n \times n}}$. Initially, the frequency matrix *FM* is calculated by random walk and on the basis of *FM* we calculate the matrix *P*. Furthermore, we define the convolution function *Conv*_*GP*_ based on *P*. We can calculate the matrix *FM* as follows: A random user can choose the random path. If the random user is at node *x*_*i*_ at time *t*, we define the state as *s*(*t*) = *x*_*i*_. We set the probability of transition from node *x*_*i*_ to one of its neighbors *x*_*j*_ by

(17) $$p\left( {s\left( {t + 1} \right) = {x_j}\left| {} \right.s\left( t \right) = {x_i}} \right) = {A_{i,j}}/\sum\limits_j {A_{i,j}}.$$

The frequency matrix is computed for all pairs of nodes and the path is calculated by random walk. The *i*^*th*^ vector of the frequency matrix is the *i*^*th*^ node and *j*^*th*^ node is the *j*^*th*^ column of the frequency matrix. This is called context *c*_*j*_. This frequency matrix is used to calculate the PPMI matrix *P*, as shown in Eqs. (18) to (21).

(18) $${p_{i,j}} = \displaystyle{{F{M_{i,j}}} \over {\sum\nolimits_{i,j} F{M_{i,j}}}},$$

(19) $${p_{i,*}} = \displaystyle{{\sum\nolimits_j F{M_{i,j}}} \over {\sum\nolimits_{i,j} F{M_{i,j}}}},$$

(20) $${p_{*,j}} = \displaystyle{{\sum\nolimits_i F{M_{i,j}}} \over {\sum\nolimits_{i,j} F{M_{i,j}}}},$$

(21) $${P_{i,j}} = \max \{ pm{i_{i,j}} = \log \displaystyle{{{p_{i,j}}} \over {{p_{i,*}}{\kern 1pt} {p_{*,j}}}},0\} ,$$

where *p*_*i*,*j*_ is the estimated probability that node *x*_*i*_ occurs in context *c*_*j*_, *p*_*i*,*_ is the estimated probability of node *x*_*i*_, and p_*,*j*_ is the probability of context *c*_*j*_, thus,

$pm{i_{i,j}} = \left\{ {\matrix{ {0,} & \ {{\rm if}\,{p_{i,j}} = {p_{i,*}} \cdot {p_{*,j}}\;({x_i}\,{\rm and}\,{c_j}\;{\rm are}\,{\rm independent});} \cr { \gt 0,} & \ {{\rm if}\,{p_{i,j}} \gt {p_{i,*}} \cdot {p_{*,j}}\;({\rm between}\,{x_i}\,{\rm and}\,{c_j}\;{\rm is}\,{\rm a}\,{\rm semantic}\,{\rm relation});} \cr { \lt 0,} & \ {{\rm if}\,{x_i}\;{\rm is}\,{\rm unrelated}\,{\rm to}\;{c_j}.} \cr } } \right.$

**Figure 7:**
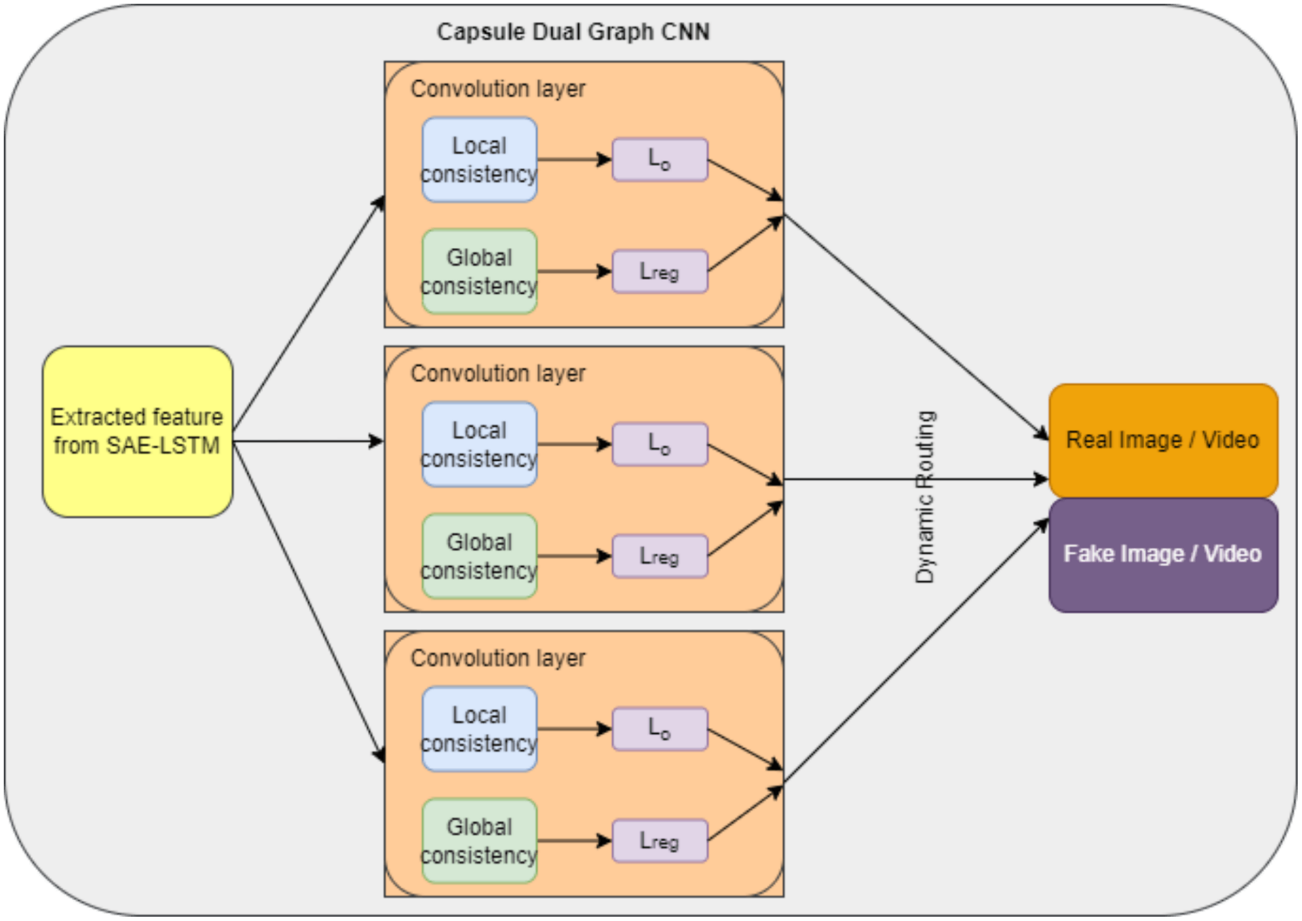
Capsule dual graph CNN structure.

The PPMI matrix *P* increases the relationship between data points compared to the adjacency matrix *A*. Using the PPMI matrix, global consistency is calculated in Eq. (22)

(22) $$Conv_{GC}^{(i)}(X) = {Z^{(i)}} = \sigma ({D^{ - \textstyle{1 \over 2}}}P{D^{ - \textstyle{1 \over 2}}}{Z^{(i - 1)}}{W^{(i)}}),$$

where *P* represents the PPMI matrix and ${D_{i,i}} = \sum\nolimits_j {P_{i,j}}$ for normalization. To combine the local and global consistency convolution for the dual graph convolutional network, a regularizer was used. The loss function with this regularizer is represented in Eq. (23)

(23) $$Loss = {L_0}\left( {Con{v_{LC}}} \right) + \lambda (t) \cdot {L_{reg}}(Con{v_{LC}},Con{v_{GC}}),$$

(24) $${L_0}\left( {Con{v_{LC}}} \right) = - \displaystyle{1 \over {|{y_L}|}}\sum\limits_{l \in {y_L}} \sum\limits_{i = 1}^c {Y_{l,i}}\log \hat Z_{l,i}^A,$$

where *c* is the number of different labels for prediction, ${Z^A} \in {{\rm {\mathbb R}}^{n \times c}}$ is the output given by *Conv*_*LC*_, ${\hat Z^A} \in {{\rm {\mathbb R}}^{n \times c}}$ is the output of the softamax layer, *y*_*L*_ represents the set of data indices whose labels are observed for training and $Y \in {{\rm {\mathbb R}}^{n \times c}}$ is the ground truth.

(25) $${L_{reg}}\left( {Con{v_{LC}},Con{v_{GC}}} \right) = \displaystyle{1 \over n}\sum\limits_{i = 1}^n {\left| {\left| {\hat Z_{i*}^P - \hat Z_{i*}^A} \right|} \right|^2},$$

where ${\hat Z^P} \in {{\rm {\mathbb R}}^{n \times c}}$ is the output of applying the softmax activation function given by *Conv*_*GC*_ (the vectors $\hat Z_{i*}^A,\hat Z_{i*}^P \in {{\rm {\mathbb R}}^n}$ are *i*^*th*^ columns of the matrices ${\hat Z^A}$, ${\hat Z^P}$, respectively). For the calculations of ${L_0}\left( {Con{v_{LC}}} \right)$ and ${L_{reg}}\left( {Con{v_{LC}},Con{v_{GC}}} \right)$, the activation function called ReLU was used. After applying the activation function, the output matrix is represented as ${Z^A} \in {{\rm {\mathbb R}}^{n \times c}}$ and ${Z^P} \in {{\rm {\mathbb R}}^{n \times c}}$. The structure of the CNN dual graph capsule is shown in Fig. 7. This proposed network consists of three main capsules. Each capsule consists of a dual graph CNN and two capsules to represent real and fake images or videos. The output of each CNN capsule called *OC*_*j*|*i*_ is directed through dynamic routing to produce the detected output *O*_*j*_ for *r* iterations, as mentioned in Algorithm 2.

**Algorithm 2:** Capsule Dual Graph CNN (C-DGCNN).

**Input:** *FM*, *A*, *PPMI*, *y*_*L*_, *r*, *λ*(*t*) and hidden convolution layers (H)
**Output:** Training model with best features.
Step 1: **for** *t* **in range** (0, Epoch number) **do**
Step 2: ${Z^A} = ReLU\left( {Con{v_{LC}}} \right)$ using Eq. (16)
Step 3: ${Z^P} = ReLU\left( {Con{v_{GC}}} \right)$ using Eq. (22)
Step 4: Compute Loss using Eq. (23)
Step 5: **if** convergence **then** break loops
Step 6: **end if**
Step 7: **end for**
Step 8: Dynamic routing procedure (*OC*_*j*|*i*_,*W*′,*r*). // Where ${W}^{\prime} \in {{\rm {\mathbb R}}^{m \times n}}$ is the matrix of wights.
Step 9: $\overline {{W}^{\prime}} \leftarrow {W}^{\prime} + rand\left( {size\left( {{W}^{\prime}} \right)} \right)$
Step 10: **for** all input capsules *i* and all output capsules *j* **do**
Step 11: $O{C_{j|i}} \leftarrow \overline {{{{W}^{\prime}}_j}}$ *squash* (*OC*_*j*|*i*_)) // Where $\overline {{{{W}^{\prime}}_j}} \in {{\rm {\mathbb R}}^m}$.
Step 12: **end for**
Step 13: **for** all input capsules *i* and all output capsules *j* **do**
Step 14: ${b_{ij}} \leftarrow 0$
Step 15: **end for**
Step 16: **for** *r* iterations **do**
Step 17: **for** all input capsules *i* **do** ${c_i} \leftarrow softmax({b_i})$
Step 18: **for** all output capsules *j* **do** ${s_j} \leftarrow \sum\nolimits_i {c_{i,j}}O{C_{j|i}}$
Step 19: **for** all output capsule networks *i* **do** ${O_i} \leftarrow squash({s_i})$ // Where $squash\left( {{s_i}} \right) = \displaystyle{{{{\left\| {{s_i}} \right\|}^2}} \over {1 + {{\left\| {{s_i}} \right\|}^2}}} \cdot \displaystyle{{{s_i}} \over {\left\| {{s_i}} \right\|}}$.
Step 20: **for** all input capsules *i* and output capsules *j* **do**
Step 21: ${b_{ij}} \leftarrow {b_{ij}} + O{C_{j|i}} \cdot {O_j}$
Step 22: **end for**
Step 23: **Return** *O*_*j*_
Step 24: **end for**

## Experimental Result and Discussions

The proposed deep learning-based deepfake detection system with an efficient feature extraction and detection process is tested with fake and real images of public datasets such as FFHQ (Li & Lyu, 2019), 100K-Faces (Li, Chang & Lyu, 2018), Celeb-DF (V2) (Kipf & Welling, 2017) and WildDeepfake. The proposed system is implemented using the machine learning library called PyTorch.

### Dataset description

#### Flickr-Faces-HQ, FFHQ

Flickr-Faces-HQ, FFHQ, is a dataset that contains a group of 70,000 face images with a high-quality resolution generated by generative adversarial networks (GANs).

#### 100K-Faces

The 100K-Faces dataset contains 100,000 unique human face images generated using StyleGAN.

#### Celeb-DF (V2)

It is a large-scale video dataset with 590 real videos of celebrities and high-quality deepfakes of 5,639 videos constructed using a synthesis process with respect to over two million frames. Real videos gathered from YouTube videos and fake videos are created by swapping each pair of faces.

#### WildDeepfake

It is a real-world deepfake detection dataset collected from the Internet. The subjects of this dataset are real and fake, and they are collected from the internet sources and consist of various scenes. Each scene consists of more persons with rich facial expressions.

#### Evaluation metrics

The proposed SAE-GLSTM-based capsule dual graph CNN deepfake detection is evaluated with various evaluation metrics, such as accuracy, sensitivity, specificity, ROC and error detection rate. The deepfake detection system is compared to standard deepfake detection approaches such as VGG19 (Zi et al., 2020; Simonyan & Zisserman, 2015; Zagoruyko & Komodakis, 2016; Sandler et al., 2018).

### Accuracy

(26) $${\rm Accuracy} = \displaystyle{{TP + TN} \over {TP + TN + FP + FN}} \cdot 100$$

### Sensitivity

(27) $${\rm Sensitivity} = \displaystyle{{TP} \over {TP + FN}} \cdot 100$$

### Specificity

It is used to evaluate the rate between true negatives (TNs) and true positives (TPs)

(28) $${\rm Specificity} = \displaystyle{{TN} \over {TN + FP}} \cdot 100$$

The comparison of the accuracy of the proposed SAE-GLSTM-C-DGCNN deepfake detection is shown in Table 3 for various datasets. With the baseline of various approaches such as VGG19, ResNet, and MobileNet, we experimented with the proposed efficient deepfake image/video feature extraction with CNN capsule dual graph CNN for various datasets. For the FFHQ datasets, existing and proposed systems obtained accuracies of 84.5%, 88.32%, 91.15%, and 96.92%. For 100K-Faces datasets, the approaches obtained the corresponding accuracies of 74.12%, 80.11%, 90.21%, and 97.15%. For the Cele-DF dataset, the accuracy values are 88.43%, 89.32%, 90.01%, and 98.12%, and for the WildDeepfake dataset, they are 89.25%, 86.52%, 96.75%, and 98.91%. The results showed that the proposed system achieved better percentage results compared to traditional deepfake detection systems.

**Table 3:** Accuracy comparison of the proposed *vs* traditional baseline systems for various datasets.

Methods	Datasets			
	FFHQ	100K-Faces	Celeb-DF	WildDeepfake
VGG19	84.5	74.12	88.43	89.25
ResNet	88.32	80.11	89.32	86.52
MobileNet	91.15	90.21	90.01	96.75
Proposed SAE-GLSTM-CDGCNN	96.92	97.15	98.12	98.91

**Figure 8:**
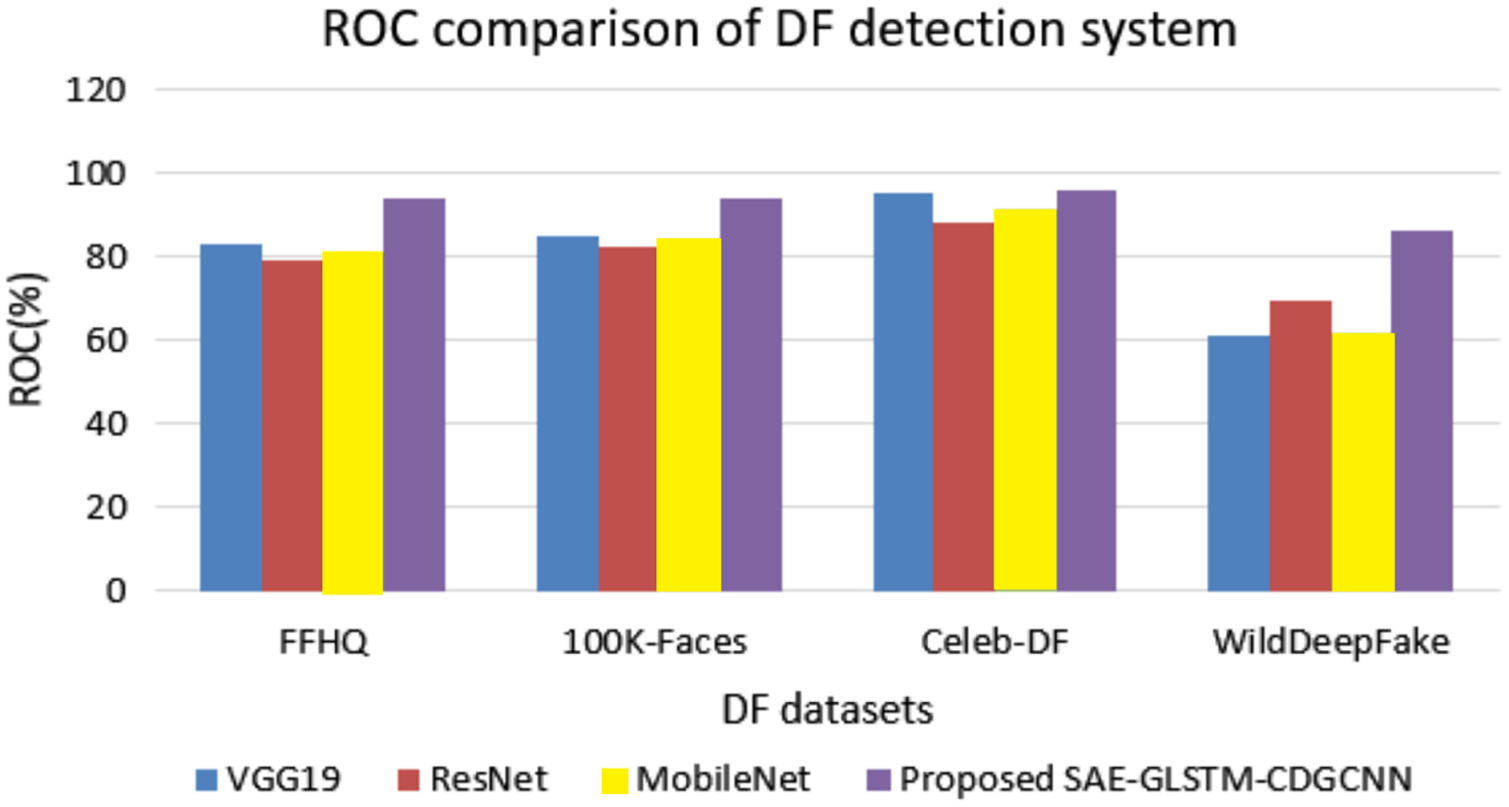
ROC value comparison.

The sensitivity, specificity, and ROC comparisons of various deepfake detection systems are evaluated using four different datasets, and the results are shown in Table 4 and Fig. 8.

**Table 4:** Sensitivity and specificity analysis of the proposed system on different datasets.

Datasets	Sensitivity %				Specificity %			
	VGG19	ResNet	Mobile- Net	Proposed SAE-GLSTM-CDGCNN	VGG19	ResNet	Mobile- Net	Proposed SAE-GLSTM-CDGCNN
FFHQ	87.3	81.6	84.2	91.67	84.23	86.1	85.2	92.4
100K-Faces	86.2	83	85.6	89.8	84.2	86.2	89.54	93.5
Celeb-DF	84.9	88.3	83.7	89.1	87.1	87.1	89.1	94.2
WildDeepfake	91.2	85.3	90.1	93.1	86.1	89.4	86.3	95.2

Table 4 shows the sensitivity and specificity analyzes of the proposed SAE-GLSTM with the C-DGCNN system compared to the existing algorithms and various datasets from FFHQ, 100K-Faces, Celeb-DF, and WildDeepfake. The proposed SAE-GLSTM with C-DGCNN obtained a sensitivity score of 91.67% in the FFHQ dataset, 89.8% in the 100K-Faces dataset, 89.1% in the Celeb-DF dataset and 93.1% in the WildDeepfake dataset. Similarly, the specificity of the proposed SAE-GLSTM with the C-DGCNN achieved a score of 92.4% in the FFHQ dataset, 93.5% in the 100K-Faces dataset, 94.2% in the Celeb-DF dataset, and 95.2% in the WildDeepfake dataset. The proposed deepfake detection of fake video/images obtained improved sensitivity and specificity percentages compared to other existing approaches. Figure 8 shows the comparison of the ROC values of various deapfake detection systems with various datasets. From the analysis, the proposed system obtained an improved ROC value of 94.2% for the FFHQ dataset, 94.1% for the 100K-faces dataset, 96.12% for the Celeb-DF dataset, and 86.54% for the WildDeepfake dataset. On the contrary, the baseline VGG19 system obtained ROC values for the FFHQ, 100K-Faces, Celeb-DF, and WildDeepfake datasets of 83.1%, 85.1%, 95.53% and 61.12%, respectively, the baseline ResNet approach secured ROC values of 79.2%, 82.3%, 88.47% and 69.78%, respectively, and the baseline MobileNet approach obtained ROC values of 81.2%, 84.1%, 91.72% and 61.54%, respectively. From the comparison, the proposed deapfake detection system secured an improved ROC value compared to traditional baseline systems.

The error detection rate comparison of the proposed *vs* existing approaches is shown in Table 4 evaluated on the FFHQ, 100K-Faces, Celeb-DF, and WildDeepfake datasets. These datasets are used in both the training and testing processes by using the deepfake detection classifier baseline methods namely, VGG19, ResNet, and MobileNet, with our proposed work of SAE-GLSTM with the C-DGCNN model. Table 5 shows that the proposed approach obtained a minimum error rate of 5.1 for the WildDeepfake dataset, 7.12 for Celeb-DF, 6.01 for 100K-Faces and 5.91 for FFHQ datasets. The error rate is minimal compared to the baseline deepfake detection methods such as VGG19, ResNet, and MobileNet.

**Table 5:** Performance comparison of the proposed methods with different datasets in terms of the error detection rate.

DF detection methods	Datasets			
	FFHQ	100K-Faces	Celeb-DF	WildDeepfake
VGG19	13.11	18.32	12.3	11.4
ResNet	13.4	15.2	12.1	11.2
MobileNet	11.2	14.22	11.65	9.21
Proposed SAE-GLSTM-CDGCNN	5.91	6.01	7.12	5.1

**Figure 9:**
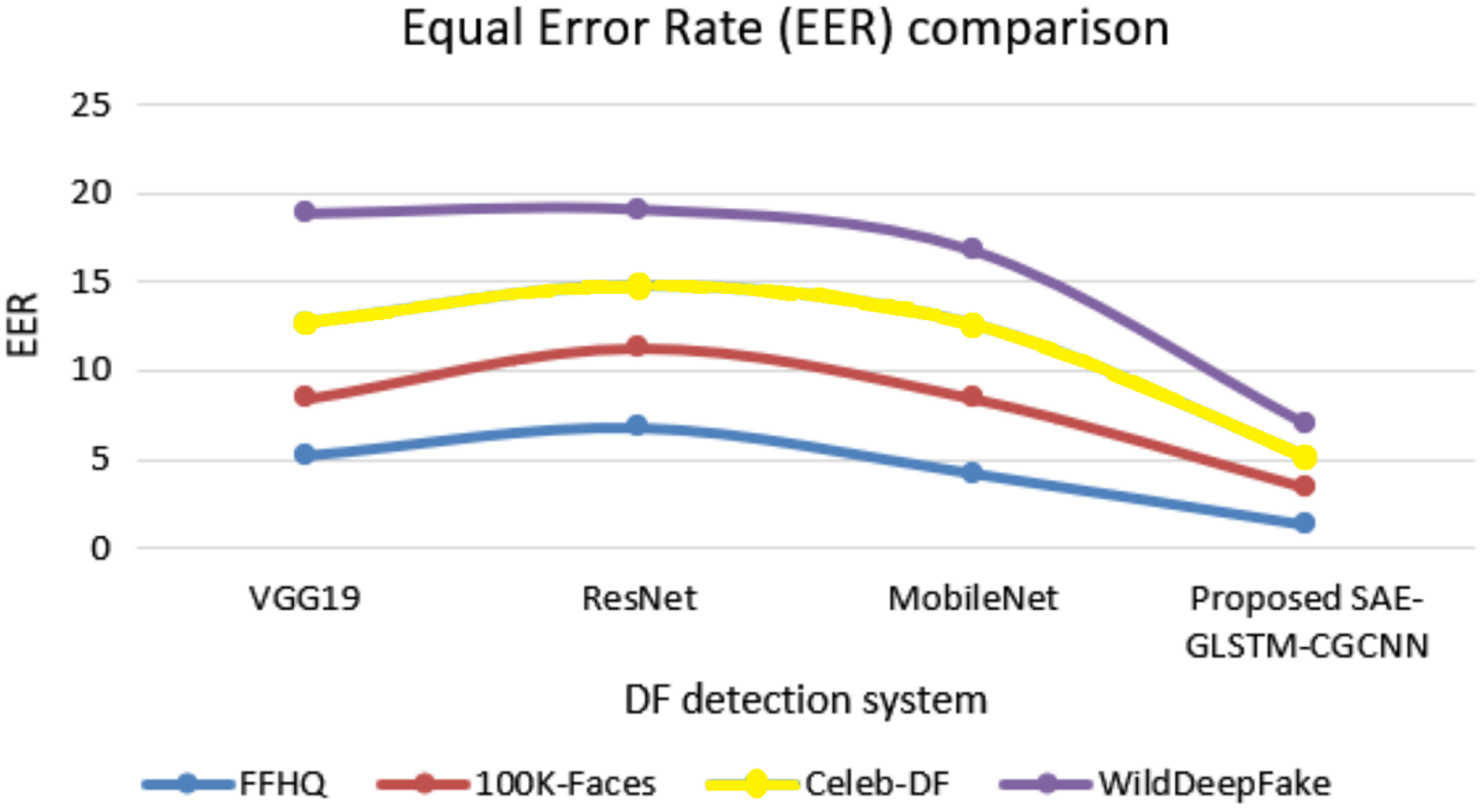
EER comparison of DF detection systems.

Figure 9 illustrates the equal error rate (EER) of various approaches with respect to various datasets. The proposed deapfake detection system secured a minimum EER of 1.9 for the WildDeepfake dataset, 1.67 for the Celeb-DF dataset, 2.1 for the 100K-Faces dataset and 1.32 for the FFHQ dataset. These EERs are minimal compared to traditional baseline systems such as VGG19, ResNet and MobileNet.

**Figure 10:**
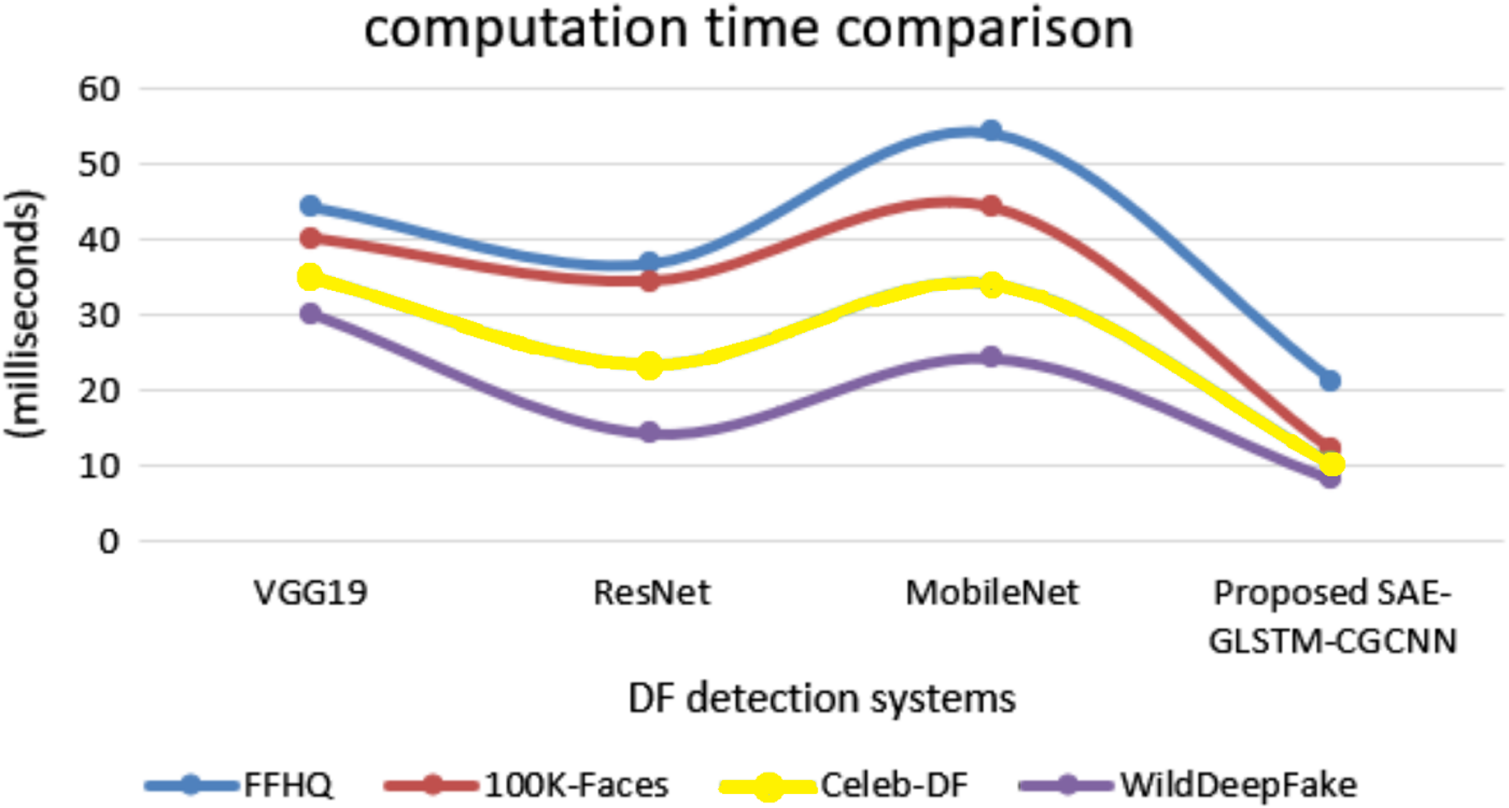
Computation time.

Figure 10 shows the comparison of computational time. The proposed system secured 8.1 ms for the WildDeepfake dataset, 10.3 ms for Celeb-DF, 12.1 ms for 100K-Faces and 21.2 ms for the FFHQ dataset. This is minimal compared to other traditional baseline deepfake detection systems, where VGG19 secured computational times for the datasets of 24.2, 34.12, 44.24 and 54.23 ms. ResNet obtained 14.22, 23.45, 34.5, and 36.81 ms, MobileNet secured 30.2, 35.1, 40.2, and 44.4 ms. Therefore, all evaluation results have shown that the proposed SAE-GLSTM with CNN capsule dual graph improved sensitivity, specificity, accuracy, and minimum error rate, EER, and computation time. Ultimately, these findings have proven that the proposed system efficiently detects deepfake images/videos with improved accuracy and minimum error.

## Conclusion

This article demonstrated the two-level deep learning method for the detection of deepfake images and videos. From the frames extracted, face images are extracted for deepfake detection. The features of the face images are extracted using the proposed SAE method. The most relevant features are extracted by enhancing the SAE-based feature extraction with the graph LSTM approach. These relevant extracted features are then fed as input into the capsule network for the detection of deepfakes. There are five capsules, including three input capsules constructed from CNN graph and two output capsules to represent fake and real images or videos. Experimental analysis with various baseline deepfake detection approaches, such as VGG19, ResNet and MobileNet, using the benchmark deepfake image and video datasets, including FFHQ, 100K-Faces, Celeb-DF and WildDeepfake, demonstrated that the proposed two-level deepfake detection approach secures improved accuracy of 96.2%, 97.15%, 98.12% and 98.91%, respectively, on these datasets. The proposed system obtained an improved ROC value of 94.2% for the FFHQ dataset, 94.1% for the 100K-Faces dataset, 96.12% for the Celeb-DF dataset and 86.54% for the WildDeepfake dataset. In terms of the error rate, the proposed system secured the corresponding values of 5.91, 6.01, 7.12 and 5.1. The proposed system secured the computational time as 8.1 ms for the WildDeepfake dataset, 10.3 ms for Celeb-DF, 12.1 ms for 100K-Faces and 21.2 ms for the FFHQ dataset. Therefore, all evaluations have shown that the proposed two-level deepfake detection method is general and effective in detecting a wide range of fake videos and image attacks. In the future, the proposed system will be improved to defend against adversarial machine attacks with enhanced capabilities.

## Supplemental Information

10.7717/peerj-cs.953/supp-1 Supplemental Information 1 Implementation.

## Additional Information and Declarations

### Data Availability

The following information was supplied regarding data availability: The data is available at GitHub: https://github.com/NVlabs/ffhq-dataset and Internet Archive: https://archive.org/download/ffhq-dataset.
